# An shRNA-Based Screen of Splicing Regulators Identifies SFRS3 as a Negative Regulator of IL-1β Secretion

**DOI:** 10.1371/journal.pone.0019829

**Published:** 2011-05-17

**Authors:** Pedro Moura-Alves, Ana Neves-Costa, Helena Raquel, Teresa Raquel Pacheco, Bruno D'Almeida, Raquel Rodrigues, Iris Cadima-Couto, Ângelo Chora, Mariana Oliveira, Margarida Gama-Carvalho, Nir Hacohen, Luis F. Moita

**Affiliations:** 1 Instituto de Medicina Molecular, Faculdade de Medicina, Universidade de Lisboa, Lisboa, Portugal; 2 Centro de Biodiversidade, Genómica Funcional e Integrativa (BioFIG), Faculdade de Ciências, Universidade de Lisboa, Lisboa, Portugal; 3 Faculdade de Medicina, Universidade de Lisboa, Lisboa, Portugal; 4 Division of Rheumatology, Allergy and Immunology, Center for Immunology and Inflammatory Diseases, Massachusetts General Hospital and Harvard Medical School, Boston, Massachusetts, United States of America; 5 Broad Institute of MIT and Harvard, Cambridge, Massachusetts, United States of America; Centre de Regulació Genòmica, Spain

## Abstract

The generation of diversity and plasticity of transcriptional programs are key components of effective vertebrate immune responses. The role of Alternative Splicing has been recognized, but it is underappreciated and poorly understood as a critical mechanism for the regulation and fine-tuning of physiological immune responses. Here we report the generation of loss-of-function phenotypes for a large collection of genes known or predicted to be involved in the splicing reaction and the identification of 19 novel regulators of IL-1β secretion in response to *E. coli* challenge of THP-1 cells. Twelve of these genes are required for IL-1β secretion, while seven are negative regulators of this process. Silencing of SFRS3 increased IL-1β secretion due to elevation of IL-1β and caspase-1 mRNA in addition to active caspase-1 levels. This study points to the relevance of splicing in the regulation of auto-inflammatory diseases.

## Introduction

The success of the vertebrate immune system relies on a remarkable potential to generate highly diverse detection, transduction and effector mechanisms in addition to the ability of individual cells to rapidly adapt and respond to changing environmental conditions [1]. Transcriptional regulation in the immune system has received the most attention in recent years, but achieving such diversity and flexibility of function requires the operation of additional mechanisms of gene regulation. Alternative Splicing (AS), which affects most of human genes and is altered in at least 15% of all point mutations causing human genetic disease [2], [3], is a potential critical mechanism for the regulation and fine-tuning of physiological immune responses [4], [5].

Our genome contains much fewer coding genes than anticipated before the completion of the human genome-sequencing project [6]. Alternative splicing permits the generation of a large array of mRNA transcripts and protein isoforms from a limited number of genes [4], [5]. By including or deleting functional protein domains it can change many protein properties including protein–protein interactions, subcellular localization, stability, DNA binding, and enzymatic properties of the dominant isoforms [7]. Two recent landmark reports estimate that 92% to 95% of all human primary transcripts can undergo alternative splicing [8], [9]. This process seems to be especially prevalent and functionally significant in the immune and nervous systems [4], [10].

Microorganisms activate the innate immune system using germline-encoded pattern-recognition receptors (PRRs). Several classes of PRRs, including Toll-like receptors, recognize distinct microbial components and directly activate immune cells, including antigen presentation cells such as dendritic cells (DCs). Exposure of immune cells to the agonists of these receptors activates the NF-κB pathway, rapidly inducing the expression of cytokines (such as IL-1β), co-stimulatory molecules and other effector molecules that are part of an effective immune response [1]. This initial response is terminated several hours later through incompletely characterized molecules and pathways [11] but that are of critical importance to avoid an excessive NF-κB signaling.

The NF-κB transcription factor has been the subject of intense study for many years, reflecting its importance in the immune response. Interestingly, many components along the pathways leading to NF-κB activation, such as MyD88, IRAK family members, TAB1, TAK1, IkB kinase complex factors and NF-κB transcription factors (reviewed in [5]) have been shown to undergo induced alternative splicing in the later phase of the immune response and to generate shorter isoforms that can act as dominant negative factors able to shut down NF-κB dependent transcription.

Here we report the use of a subset of the TRC lentiviral human library [12] to generate loss-of-function phenotypes for a large collection of genes known or predicted to be involved in the splicing reaction (425 genes), with an average 5-fold coverage [13]. By applying this library in human THP-1 monocytic cells, we aim to systematically identify components of the splicing machinery that modulate the secretion of IL-1β. We have identified 19 genes that significantly affect the production of IL-1β after a 24 h challenge with PFA-fixed *E. coli*. Twelve of these genes are required for IL-1β secretion, while seven are negative regulators of this process. We chose one of the highest scoring negative regulators, SFRS3, for further characterization of its role in IL-1β secretion.

## Methods

### Cell Culture

THP-1 cells (ATCC TIB-202) were grown in R10- RPMI media 1640 supplemented with 10% (v/v) Fetal Bovine Serum, 1% (v/v) Penicillin-Streptomycin, 1% (v/v) Pyruvate , 1% (v/v) L-Glutamine , 1% (v/v) Non-essential aminoacids , 1% (v/v) Hepes buffer and 0.05 M of 2-Mercaptoethanol, HEK 293T cells (ATCC CRL-11268) were grown in Dulbecco's modified Eagle's medium (DMEM) supplemented with 10% (v/v) Fetal Bovine Serum, 1% (v/v) Penicillin-Streptomycin and 0.05 M of 2-Mercaptoethanol. Cells were kept at 37°C under a 5% carbon dioxide (CO_2_) atmosphere. Purification of bone marrow derived cells (BMDCs) was adapted from previously described [14], [15]. Briefly, marrow cavities of the tibias and femurs of 8–12 week-old mice were flushed with complete RPMI 1640 (10% FBS) using a 27-gauge needle (Terumo, Tokyo, Japan). After red cells hypotonic lysis for 5 min (with ammonium chloride solution), BM cells were washed and seeded in 96 well round bottom plates (5×10^4^/well), in culture medium supplemented with 30% conditioned medium from mouse granulocyte-macrophage colony stimulating factor (GM-CSF) producing J558L cells. Peripheral blood mononuclear cells (PBMCs) were isolated from buffy coats of healthy donors by density gradient centrifugation using Ficoll-Paque (GE Healthcare). Monocytes were magnetically isolated from PBMCs with CD14 microbeads (Miltenyi Biotec, Germany).

### The RNAi Consortium RNAi library and lentiviral infection

Detailed description of the RNAi Consortium (TRC) lentiviral RNAi library used in this study was originally described in [13] and [16]. Briefly, most splicing and NLR genes were targeted with ∼5 short hairpin RNAs (shRNAs) expressed under the control of the U6 Pol III promoter in a lentiviral vector (pLKO.1) that also confers puromycin resistance. Plasmid DNA purification, lentiviral production and infection were performed as described [12] (and see www.broad.mit.edu/rnai/trc/lib for additional details).

### IL-1β secretion assay

THP-1 cells were infected with the shRNA-expressing lentivirus and selected using puromycin 48 hrs later. After the 3 days of selection, plates were duplicated. One of the plates was used to measure the cell number using Alamar Blue cell viability assay (Invitrogen), according to manufacturer's instructions. In the other plate, cells were stimulated with 4% PFA-fixed DH5α *E. coli* at a Multiplicity of Infection (MOI) of 20 bacterial cells per THP-1 cell. After the indicated times, cell supernatants were collected and IL-1β cytokine quantified by ELISA.

### ELISA, qRT-PCR, IB and FACS

Cytokine concentrations in cell supernatants were assayed by ELISA using Human IL-1 beta/IL-1F2 DuoSet (R&D Systems), according to company's protocol. For quantitative RT-PCR (qRT-PCR), **t**otal RNA was extracted using TRIzol reagent (Invitrogen) and cDNA synthesis used Superscript II Reverse Transcriptase (Invitrogen). qRT-PCR was performed in the presence of Power SYBR green PCR Master Mix (Applied Biosystems) and the amplification protocol was performed on a Rotor-Gene 6000 (Corbett). All samples were normalized to the expression of glyceraldehyde-3-phosphate dehydrogenase (GAPDH) and relative expression was calculated using *Pfaffl*'s method [17].

For immunoblotting (IB), cells were collected, washed twice and lysed for 15 minutes at 4°C using RIPA buffer (50 mM Tris-HCl at pH = 7.4, 1% NP-40, 0.25% Sodium Deoxicholate, 150 mM NaCl, 1 mM EDTA, 1 mM Na_3_VO_4_, 1 mM NaF in the presence of proteases inhibitor cocktail (Roche)). Primary antibodies against β-actin, caspase-1, HMGB1, NLRP3 and V5 were from Abcam; against ASC and SFRS3 were from Abnova. Secondary antibodies against mouse or rabbit were from Cell Signaling. Caspase-1 activity was measured using the Carboxyfluorescein FLICA Detection kit for Caspase Assay (Immunochemistry Technologies, LLC). Briefly, cells were incubated for 1 hour at 37°C with 30× FLICA solution at a 1∶30 ratio, washed 3 times, and ressuspended in 150 µL of wash buffer. The samples were immediately assayed by Flow citometry, using a FACSCalibur system (BD biosciences) and the data was analysed using FlowJo software (Tree Star Inc).

### SFRS3 Overexpression

Lentiviral vector for SFRS3 protein overexpression was constructed using the Gateway pENTR11 and pLenti6.2/V5-DEST Gateway vectors from Invitrogen. SFRS3 was amplified from THP-1 cells cDNA.

### Data analysis

Hit identification: Values were normalized by dividing the amount of secreted IL-1β in the conditioned media 24 hrs after *E. coli* stimulation by the number of cells in each well and then by the average concentration per cell of the plate. Results were logarithmic natural transformed. Scores were sorted in ascending order and graphed. We calculated 1.5 SDEVs above and below the average and identified genes for which at least two independent targeting shRNAs gave similar phenotypes. After two or more rounds of phenotypic validation, we chose 30 genes that reproducibly changed IL-1β secretion when silenced. Statistic analysis: The data were compared using the t-student test, and analyzed using the Prism software unless stated otherwise. All data presented are the average of a minimum of three independent biological replicas.

### NF- κB reporter gene assay

Lentiviral particles carrying a NF-κB-responsive GFP-expressing reporter gene (Cignal Lenti Reporters, SABiosciences) were used to infect THP-1 cells and to establish a stable cell line. For the infections, 10 µL of lentiviral particles were used and the protocol was according to the manufacturer. Once established, the THP-1 NF- κB responsive cell line was infected with lentivirus expressing shRNAs for the specific silencing of SFRS3. For each infection, 20 µL of shRNA lentivirus were used; otherwise the protocol was as described above, including the infectious stimulus with PFA-fixed *E. coli*. The GFP fluorescence was assayed by flow cytometry using a FACSCalibur system (BD biosciences).

### Actinomycin D treatment

THP-1 cells infected with shRNA-expressing lentivirus were stimulated at day six of infection with PFA-fixed *E. coli* at MOI of 20 bacteria per cell. Two hours post stimulation, the cells were treated with 5 µg/mL of actinomycin D (ActD, AppliChem) and incubated for 1, 2, 3 or 4 hours, after which were collected for RNA extraction. The half-life (t1/2) of IL-1β mRNA was determined by adjusting an exponential trendline to the data points and using the formula t1/2 = ln(2)/b (b obtained from the exponential function y = c*e* ^bx^).

### IL-1β primers

Exonic IL-1β primers: CTCGCCAGTGAAATGATGGCT (forward) and GTCGGAGATTCGTAGCTGGAT (reverse). Intronic IL-1β primers: TCACACGGAAAGTTGGGGGCC (forward) and TGTGGGGCAAGGGACAAAGATG (reverse). All other primers used in qRT-PCR were obtained from Primer Bank http://pga.mgh.harvard.edu/primerbank/index.html.

## Results

### THP-1 cells secrete IL-1β in response to *E. coli* challenge

**Figure 1:**
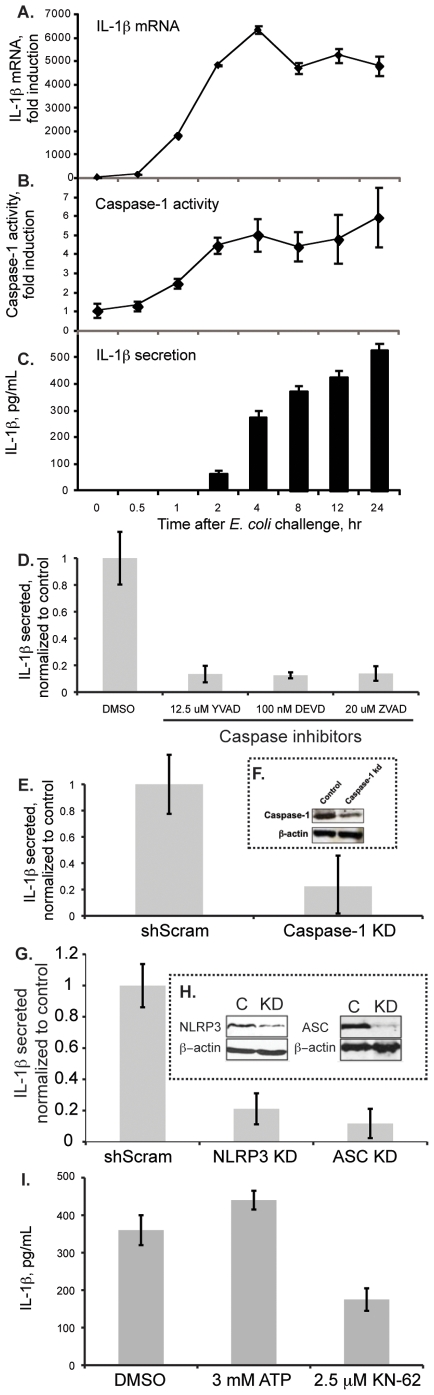
THP-1 cells induce and secrete IL-1β in response to *E. coli* stimulation. (A) THP-1 cells were challenged with PFA-fixed *E. coli* at a 20 bacteria: 1 cell ratio. IL-1β mRNA expression was quantified by qRT-PCR; (B) Caspase-1 activity as measured by FACS using fluorescent caspase-1 substrate (FLICA); (C) In the same conditions, cell conditioned media was collected and the concentration of IL-1β measured by ELISA; (D) Caspase-1 dependent IL-1β secretion at 24 hrs after *E. coli* challenge is shown by using caspase drug inhibitors; (E) and by measuring IL-1β secretion from cells where caspase-1 was depleted by RNAi; (F) validation of caspase-1 silencing using immunoblotting; (G) Effect of NLRP3 and ASC in IL-1β secretion after 24 hrs of *E. coli* challenge; (H) Validation of NLRP3 and ASC knockdowns, by immunoblotting using specific antibodies against these inflammasome components; (I) the secretion of IL-1β was compared in THP-1 cells that were stimulated with PFA-fixed *E. coli* with cells that in addition to *E. coli* stimulation were treated with either ATP or the P2X7 inhibitor, KN-62.

The sequence of events culminating in IL-1β secretion is complex, but can be summarized in two steps: induction of pro-IL-1β and its processing by activated caspase-1 (reviewed in [18]). The human monocytic cell line THP-1 has been a preferred *in vitro* model system to study the mechanisms of IL-1β induction and processing [19]. To search for novel regulators of IL-1β secretion, we begun by the development and characterization of an *in vitro* assay to systematically test the role of splicing factors and regulators of splicing in this process. Most of the available studies have used purified pathogen associated molecular patterns (PAMPs) to induce IL-1β message transcription and ATP for the triggering of inflammasome activation to drive its processing (reviewed in [20]). We have challenged THP-1 cells with LPS in the absence of ATP over a 24 hr period. Under these conditions we have observed no significant secretion of IL-1β (D'Almeida, unpublished), confirming previous observations (reviewed in [21]). However, stimulation of THP-1 cells with PFA-fixed *E. coli* caused significant IL-1β mRNA induction (Figure 1A), caspase-1 activity (Figure 1B) and IL-1β secretion (Figure 1C), in a range of concentrations comparable to previous reports [22].

Stimulation of THP-1 cells with PFA-fixed *E. coli* caused a dramatic increase in IL-1β mRNA expression as measured by qRT-PCR (Figure 1A). Upon challenge, the levels of IL-1β mRNA rose quickly and peaked at 4 hr after which they remained high for the duration of the assay. We have used ELISA to measure the concentrations of IL-1β in the corresponding conditioned media. IL-1β secretion can be detected already at 2 hr after bacterial challenge, but we observe a major increase at 4 hr, after which the concentration of IL-1β steadily increases and only stabilizes at 48 hr after stimulation (Figure 1C and data not shown). Interestingly, caspase-1 activity, as measured by FACS using a caspase-1 fluorescent substrate that irreversibly binds to its catalytic site, progressively increases after *E. coli* challenge and peaks at 4 hrs after which it remains stable, with a modest increase after 12 hrs (Figure 1B).

Most of the IL-1β processing activity is mediated by caspase-1, but other proteases can play a role (reviewed in [23]). In our system, most of the IL-1β processing activity is accomplished by caspase-1. In fact, broad caspase inhibitors (Z-VAD and Z-DEVD) and the more specific caspase-1 inhibitor Z-YVAD almost completely block the secretion of IL-1β (Figure 1D). In addition, the specific depletion of caspase-1 from THP-1 cells using RNAi has a similar effect (Figure 1E and F). Together, the drug and RNAi inhibition of caspase-1 results, show caspase-1-dependent secretion of IL-1β, in our assay.

To identify the dominating inflammasome components driving IL-1β secretion in our assay, we have performed a small-scale screen covering most of the NLR and CARD domain containing proteins. We found roles for several inflammasome components that significantly affect IL-1β secretion after 24 hr of *E. coli* challenge. Silencing of NLRP3 and ASC caused the strongest phenotypes (Figure 1G, and Moura-Alves, unpublished). The depletion of NLRP3 and ASC were validated at the protein level using immunoblotting (Figure 1H).

Previous studies have documented the requirement for ATP in the processing and secretion of IL-1β [24]. However, we observe high levels of secretion of IL-1β without addition of exogenous ATP to *E. coli* challenged THP-1 cells (Figure 1C). To check if the addition of ATP is critical in our system, we supplied ATP to *E. coli* challenged THP-1 cells, but found only a marginal increase in the level of secreted IL-1β (Figure 1I). Interestingly, by using a known inhibitor of the purinergic receptor P2X7 (KN-62) [25], we observed a significant decrease in the concentration of secreted IL-1β (Figure 1I). We conclude that ATP is required for the processing and secretion of IL-1β in our assay, but that its exogenous supply is not necessary, because it is available in culture possibly resulting from a small percentage of dying cells in response to *E. coli* challenge or active secretion which can activate THP-1 cells in an autocrine way [26].

### An shRNA-based screen identifies splicing factors and regulators of splicing with a role in IL-1β secretion

To identify splicing regulators with a role in IL-1β secretion by THP-1 cells after *E. coli* challenge, we used a subgenomic library targeting 425 genes (Supplementary Table S1) that was recently used successfully for the identification of hnRNPLL as a master regulator of CD45 alternative splicing [13]. This subset of the RNAi Consortium (TRC) library (www.broad.mit.edu/rnai/trc/lib) is enriched on splicing factors (SFs) and regulators of splicing and has been assembled based on reported proteomic, bioinformatics and genetic studies for splicing factor identification and spliceosome composition characterization [27], [28], [29], [30], [31]. A complete list of the targeted SFs can be found in supplementary materials.

**Figure 2:**
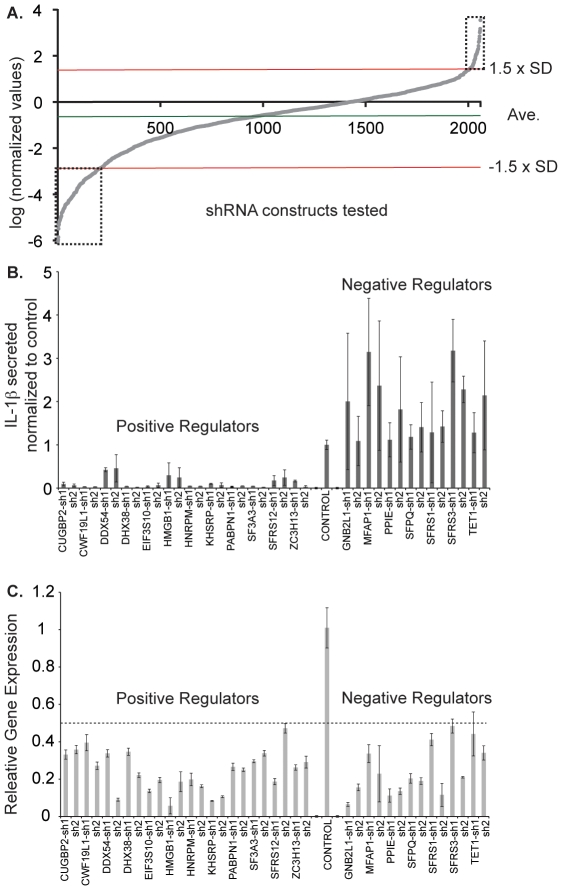
Identification and validation of novel splicing factors with a role in IL-1β secretion. (A) THP-1 cells were infected with shRNA expressing lentiviruses in a 96-well plate format. After puromycin selection, the number of cells was measured and stimulated with PFA-fixed *E. coli*. Values of secreted IL-1β were normalized by dividing the amount of IL-1β in the conditioned media 24 hrs after *E. coli* stimulation by the number of cells in each well and then by the average concentration per cell of the plate. Results were logarithmic natural transformed. Scores were sorted in ascending order and graphed; (B) Phenotypes for 12 positive regulators and 7 negative regulators of IL-1β secretion are shown in comparison to shScram control; (C) Validation of candidates by measurement of mRNA levels after silencing using qRT-PCR.

THP-1 cells were infected with lentivirus particles expressing individual short hairpin RNAs (shRNAs) and selected with puromycin 48 hrs later. Six days after infection, cells were split into two plates. One was used to measure the number of cells. All wells with less than 10.000 cells were excluded from analysis, because we found that bellow this cut-off there is high variability in cytokine secretion with different bacterial amounts (data not shown). In the other plate, cells were stimulated with PFA-fixed *E. coli* at a ratio of 20 bacteria per cell. ELISA was used to measure the resulting concentrations of IL-1β in the conditioned media 24 hrs after *E. coli* stimulation. The concentrations of IL-1β were divided by the number of cells in each well and again divided by the mean concentration per cell of the plate. Increases and decreases in relation to plate average are better identified from a log transformation of normalized (N) values that appropriately fits the data points in a linear relationship [32]. Accordingly, we did a logarithmic natural transformation and graphed the sorted scores in ascending order (Figure 2A). To quantify the deviation of cytokine levels from the mean of all measurements within the same plate, we calculated 1.5 SDEVs above and below the mean and identified genes for which at least two independent targeting shRNAs gave similar phenotypes. After two or more rounds of phenotypic validation, we chose 30 genes that reproducibly changed IL-1β secretion when silenced. Twenty genes decreased the levels of IL-1β secretion when silenced (positive regulators), while 10 led to significant increase of IL-1β secretion after depletion (negative regulators).

We then used qRT-PCR to correlate the efficacy of knockdown with the phenotypes observed. For 19 out of the 30 initial candidates we found a good correlation between the best two shRNAs in each gene and the degree of knockdown for the same hairpins (Figure 2C). Twelve of the validated genes are positive regulators (Table 1) and seven are negative regulators (Table 2).

**Table 1:** IL-1β secretion candidate positive regulator genes.

Gene ID	Symbol	Description/Role
10659	Celf2	Pre-mRNA alternative splicing and may also be involved in mRNA editing, and translation
55280	CWF19L1	NA
79039	DDX54	Translation initiation, nuclear and mitochondrial splicing, and ribosome and spliceosome assembly.
9785	DHX38	Translation initiation, nuclear and mitochondrial splicing, and ribosome and spliceosome assembly.
8661	EIF3A	Eukaryotic translation initiation factor 3, subunit A
3146	HMGB1	High-mobility group box 1
4670	HNRNPM	Pre-mRNA processing and other aspects of mRNA metabolism and transport
8570	KHSRP	Transcription, alternative pre-mRNA splicing, and mRNA localization.
8106	PABPN1	Polymerization of poly(A) tails on the 3′ ends of eukaryotic genes
10946	SF3A3	Pre-mRNA splicing
140890	SFRS12	Serine/arginine-rich (SR) splicing factor. Modulates splice site selection.
23091	ZC3H13	NA

**Table 2:** IL-1β secretion candidate negative regulator genes.

Gene ID	Symbol	Description / Role
10399	GNB2L1	Guanine nucleotide binding protein (G protein), beta polypeptide 2-like 1
4236	MFAP1	Microfibrillar-associated protein 1
10450	PPIE	Catalyze the cis-trans isomerization of proline imidic peptide bonds in oligopeptides and accelerate the folding of proteins.
6421	SFPQ	Transcription and RNA processing.
6426	SFRS1	Serine/arginine-rich (SR) splicing factor. Modulates splice site selection.
6428	SFRS3	Serine/arginine-rich (SR) splicing factor. Modulates splice site selection.
80312	TET1	Epigenetic regulation by modification of 5 mC to hmC

### Selection and validation of SFRS3 as a novel negative regulator of IL-1β secretion

**Figure 3:**
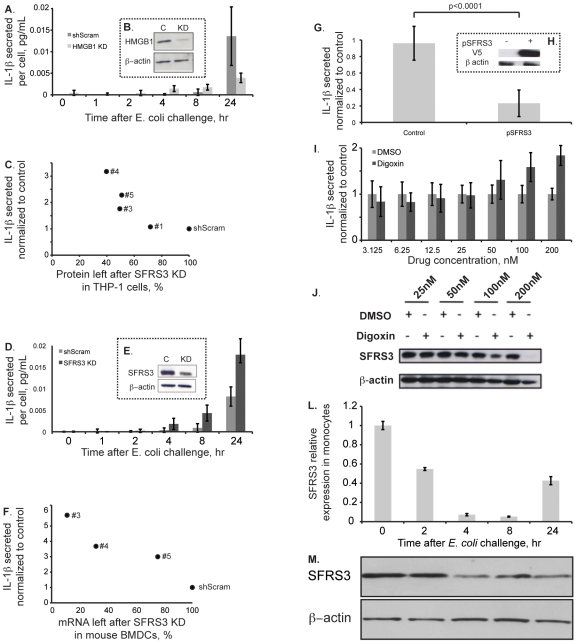
Validation of SFRS3 as a novel regulator of IL-1β secretion. (A) Kinetics of IL-1β secretion in HMGB1 KD cells after *E. coli* stimulation in comparison to the shScram control; and (B) validation of HMGB1 KD by immunoblotting; (C) SFRS3 protein left after SFRS3 silencing in THP-1 cells is plotted against the phenotype for each construct as measured by IL-1β secretion, construct #2 is not shown because most cells died; (D) Secretion of IL-1β in SFRS3 KD cells after *E. coli* stimulation in comparison to the shScram control; and (E) validation of SFRS3 KD by immunoblotting; (F) SFRS3 mRNA left after SFRS3 silencing in mouse bone marrow-derived dendritic cells is plotted against the phenotype for each construct as measured by IL-1β secretion, constructs #1 and #2 are not shown because most cells died; (G) IL-1β secretion after *E. coli* stimulation in THP-1 cells over-expressing SFRS3; and (H) validation of SFRS3 over-expression by immunoblotting using a specific antibody against the V5 tag; (I) IL-1β secretion by THP-1 cells after 24 hrs of *E. coli* challenge in the presence of growing concentrations of the SFRS3 depleting drug digoxin; (J) validation of SFRS3 depletion by immunoblotting using a specific antibody against SFRS3. SFRS3 levels are down modulated in monocytes in response to an *E. coli* challenge. (L) Kinetics of SFRS3 mRNA expression in monocytes purified from Human PBMCs after *E. coli* stimulation; (M) SFRS3 protein levels in the same conditions, by immunoblotting using a specific antibody against SFRS3.

We found the High Mobility Group Box 1 (HMGB1) gene as a positive regulator of IL-1β secretion (Figure 2B). HMGB1was included in the group of genes with a documented or possible role in splicing to test in our assay because it was identified in one of the proteomic studies of the spliceosome used to assemble the SF collection [27]. While its role in splicing has not been addressed, its identification in our study supports the power of our approach because HMGB1 has a well-documented role in the inflammatory response (reviewed in [33]) and was previously shown to regulate IL-1β secretion by the transactivation of its promoter [34]. We did a kinetic study of IL-1β secretion in THP-1 cells where HMGB1 was depleted and compared it to the results for the control cells treated with the control shRNA (shScram) and observed a strong decrease in the amount of IL-1β secreted per cell at 24 hrs after *E. coli* stimulation (Figure 3A). The shRNA tested was chosen after validating the knockdown of HMGB1 at the protein level using immunoblotting (Figure 3B).

Effective down-modulation of immune responses is critical for physiological immune responses and to avoid autoimmunity and autoinflammatory diseases such as those caused by deregulated increased secretion of IL-1β due to inflammasome hyper-activation [35]. Termination of a signal can be as important as its initiation. However, negative feedback pathways are much less well understood in immune signaling. We therefore decided to choose one of the identified negative regulators in our screen to further characterize its role in IL-1β secretion. SFRS3 was one of the candidates that showed the strongest and most consistent phenotype. Silencing of SFRS3 in the original primary screen by 3 independent shRNA constructs led to increase in the levels of secreted IL-1β above the defined cut-off. After phenotypic validation with independently produced batches of lentiviruses, we then correlated the levels of SFRS3 silencing at the protein level with the observed phenotypes for each construct (Figure 3C). Construct #2 produced the best silencing, but also caused elevated cell death possibly reflecting the essential role of SFRS3. We therefore did not include it in Figure 3C. From the plot we can clearly see that the level of SFRS3 depletion correlated with the strength of the phenotype (Figure 3C). We conclude that SFRS3 silencing is the target responsible for the increase in secreted IL-1β.

SFRS3 is a member of the SR protein family [36]. These are required for constitutive pre-mRNA splicing but also regulate alternative splice site selection in a concentration-dependent manner in addition to other documented roles in transcription, nucleo-cytoplasmic transport and translation (reviewed in [36]). Kinetic studies of IL-1β secretion in SFRS3 knockdown cells compared with cells treated with the shRNA control (Figure 3D) after the identification and validation of the best shRNA targeting SFRS3 (Figure 3C and E) show that already at 4 hrs after *E. coli* challenge there is an increase in cytokine secretion and that the difference becomes more pronounced at 8 hrs and reaches a maximum at 24 hrs post-challenge (Figure 3D).

To further validate the role of SFRS3 as a novel regulator of IL-1β secretion, we took three approaches: (1) test the role of SFRS3 in IL-1β secretion in mouse cells; (2) over-expression of SFRS3 in THP-1 cells; and (3) depletion of SFRS3 in THP-1 cells using the drug digoxin [37].

We have used 5 independent constructs to silence SFRS3 in bone marrow-derived dendritic cells (BMDCs). Two of them (#1 and #2) caused high levels of cell death. Using qRT-PCR to measure the percentage of SFRS3 mRNA left in BMDCs after treatment with constructs #3, #4 and #5, we observed an excellent correlation with the increase in the amount of secreted IL-1β after bacterial challenge of BMDCs (Figure 3F).

In accordance with the identified negative regulator phenotype, we would expect that the over-expression of SFRS3 would either produce no change in the levels of IL-1β secretion or, more convincingly, decrease cytokine secretion. In fact, over-expression of SFRS3 causes a dramatic decrease in the levels of IL-1β secretion after 24 hrs of THP-1 cell challenge with PFA-fixed *E. coli* (Figure 3G). Over-expression of SFRS3 was confirmed using immunoblotting with an antibody against the V5 tag that was fused to this factor (Figure 3H).

In a complementary approach, we used the recently identified depletion effect of digoxin on SFRS3 [37], to independently test the role of this SF on IL-1β secretion. We can show that increasing concentrations of digoxin, starting at 50 nM, directly correlate with increased amounts of IL-1β in THP-1 cell conditioned media after 24 hrs of *E. coli* stimulation (Figure 3I). Interestingly, we start observing depletion of SFRS3 with digoxin concentrations above 50 nM, which peaks at 200 nM (Figure 3J) after which we observe toxic effects to the cells (data not shown).

### SFRS3 mRNA and protein levels are regulated in monocytes in response to *E. coli* stimulation

Expression levels of another member of the SR protein family, SFRS1, are modulated by pro-inflammatory stimuli and in autoimmunity [38]. We tested the regulation of the levels of SFRS3 mRNA and protein in response to bacteria. To monitor SFRS3 mRNA and protein levels after an *E. coli* challenge in primary cells that are directly relevant for *in vivo* human production of IL-1β in health and disease, we used freshly isolated monocytes from PBMCs of volunteers and challenged them with PFA-fixed *E. coli*. We found that SFRS3 mRNA was decreased after 2 hrs of stimulation and that this tendency was more preeminent at 4 and 8 hrs post-challenge (Figure 3L). At 24 hrs, we observed an increase, suggesting a recovery of its levels at later periods (Figure 3L). At the protein level, we observed a compatible trend with delayed kinetics (Figure 3M). The decrease of SFRS3 is evident at 4 hrs and the protein levels remain low at 24 hrs after stimulation (Figure 3M). While similarly robust regulation was not observed in THP-1 cells, our reproducible findings in primary monocytes suggest that SFRS3 might play a physiologic role in the secretion of IL-1β, and that its down-regulation might be required for stronger caspase-1 activation.

### SFRS3 silencing increases IL-1β mRNA and active caspase-1 levels

The secretion of bioactive IL-1β is a two-step process. The first is the induction of pro-IL-1β that starts with the NF-κB dependent transcription of IL-1β mRNA. The second is the processing of the inactive 31 kDa pro-molecule by activated caspase-1.

**Figure 4:**
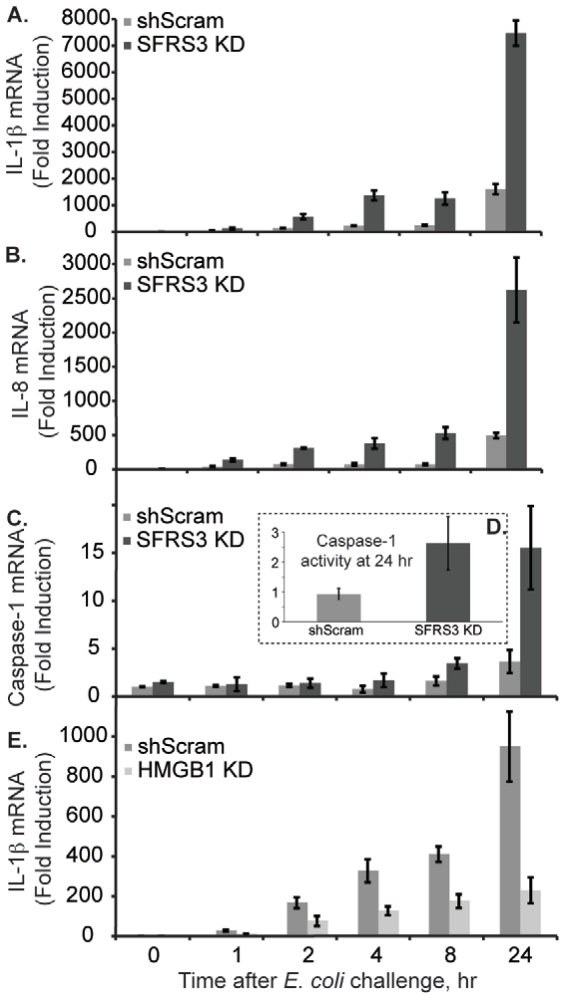
SFRS3 depletion increases IL-1β mRNA and active caspase-1 levels. (A) Comparison of the kinetics of IL-1β mRNA induction after *E. coli* challenge in SFRS3 depleted cells and control interfered THP-1 cells; (B) Comparison of the kinetics of IL-8 mRNA induction after *E. coli* challenge in SFRS3 depleted cells and control interfered THP-1 cells; (C) the same for caspase-1 mRNA; and (D) measurement of active caspase-1 by FACS using fluorescent caspase-1 substrate (FLICA); (E) Kinetics of IL-1β mRNA induction after *E. coli* challenge in HMGB1 depleted THP-1 cells.

The activation of the NF-κB pathway usually starts with the engagement of a TLR receptor, while the second step follows inflammasome activation, which can be initiated by the activation of the purinergic P2X7 receptor in response to the presence of ATP (reviewed in [20]). To understand the mechanism of regulation of IL-1β secretion by SFRS3, we silenced SFRS3 and studied its kinetic effect on the levels of IL-1β mRNA (Figure 4A), IL-8 mRNA (Figure 4B), caspase-1 mRNA (Figure 4C) and resulting caspase-1 activity at 24 hrs (Figure 4D).

We conclude that SFRS3 silencing causes an early increase in IL-1β (Figure 4A) and IL-8 mRNA (Figure 4B) which is especially significant at 24 hrs after *E. coli* stimulation. The knockdown of SFRS3 also increases the levels of caspase-1 mRNA, most preeminently at 24 hrs after challenge (Figure 4C). The increase in caspase-1 mRNA translates in an ∼2.5-fold increase in the levels of active caspase-1 at 24 hrs after stimulation, as measured by FACS using a caspase-1 fluorescent substrate (Figure 4D). We notice that the increase in caspase-1 mRNA and activity at 24 hrs in SFRS3 knockdowns correlates well with the highest difference between control and SFRS3 knockdown when measuring IL-1β secretion (Figure 3C). We conclude that SFRS3 depletion affects and increases the activity of both steps required for IL-1β secretion. As a control, we also looked at the effect of silencing HMGB1 on IL-1β mRNA levels. As expected, we observed significant and progressive differences in the levels of IL-1β mRNA, which is decreased in HMGB1 knockdown (Figure 4E).

### SFRS3 negatively regulates IL-1β transcription

**Figure 5:**
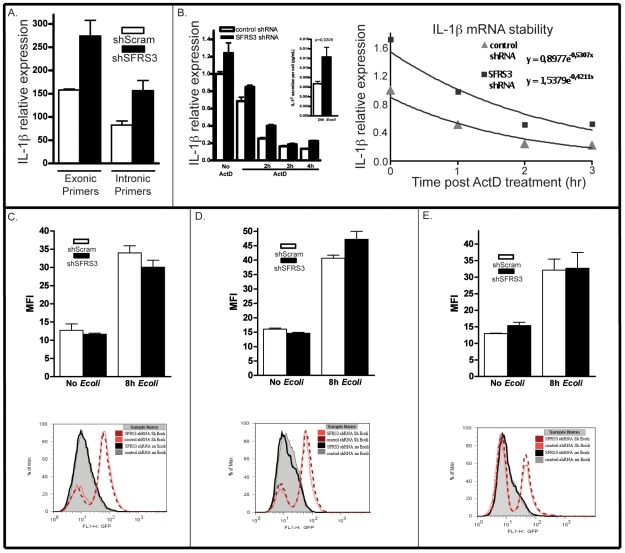
SFRS3 regulates IL-1β transcription in the context of unchanged NF- κB reporter activity. (A) IL-1β mRNA as determined using exonic or intronic primers after 8 hours of *E. coli* challenge. One representative experiment is shown; (B) Plot begins after 2 hours of *E. coli* challenge. Time on the x-axis indicates the period of Actinomycin D treatment. One representative experiment is shown. On the right, phenotypic control for this experiment as measured by IL-1β secret ion is shown (triplicates with standard deviations are shown for three independent measurements); (C, D, E) three independent experiments of NF-κB reporter activation on control or SFRS3 shRNA treated cells as measured by FACS and expressed in Mean Fluorescence Intensity (MFI). For each figure/experiment, triplicates with standard deviations are shown for three independent measurements and one representative measure for each sample is plotted bellow.

The results described in the previous section suggest that the silencing of SFRS3 could affect the level of IL-1β transcription. To directly test this hypothesis, we have used exonic and intronic primers to compare the levels of mRNA induction in control (shScram) and SFRS3-silenced cells (shSFRS3) after *E. coli* challenge as measured by qRT-PCR. We could observe that the shSFRS3 to shScram ratios of IL-1β mRNA were similar when using either exonic or intronic primers (Figure 5A), suggesting that in fact silencing SFRS3 increases the level of IL-1β transcription.

Post-transcriptional control mechanisms have been shown to affect the expression of pro-inflammatory mediators by regulating the stability and translation of the mRNAs encoding them (reviewed in [39]). To test for possible additional effects of SFRS3 at the level of the regulation of IL-β mRNA stability, we challenged THP-1 cells with PFA-fixed *E. coli* for 2 hrs that were infected with either the shRNA control or the shRNA targeting SFRS3. Cells were then treated with Actinomycin D to inhibit transcription and the levels of mRNA for IL-β (Figure 5B) were monitored by qRT-PCR at the indicated time points. Based on the results we have calculated the half-life of IL-β mRNA in control and SFRS3-silenced THP-1 cells. In three independent experiments, of which we show one representative experiment in Figure 5B, we did not observe a significant change in the half-life of IL-β mRNA caused by silencing of SFRS3, and therefore conclude that the observed phenotype is mostly due to changes in IL-β at the transcriptional level.

The transcription of IL-1β is mostly dependent on activation of the NF-κB pathway. To compare NF-kB activation, in response to *E. coli* challenge, in control THP-1 cells with those where SFRS3 was silenced, we created an NF-κB reporter cell line by stably infecting THP-1 cells with a commercial lentiviral GFP reporter under the control of a minimal CMV promoter and tandem repeats of the NF-κB transcriptional response element (TRE). Surprisingly, silencing of SFRS3 in this cell line (Figure 5C,D and E) did not change the levels of GFP reporter expression after treatment with PFA-fixed *E. coli* as compared to the shScram treated cells.

## Discussion

Alternative splicing plays an important role in the regulation of NF-κB activation by generating different isoforms for key signaling components along this pathway [5]. Using a lentiviral-based system to express shRNA we were able to generate loss-of-function phenotypes for most of the previously identified factors with a known or putative role in splicing. In our screen, we have validated 19 novel regulators of IL-1β secretion by THP-1 cells after challenge with *E. coli*. Twelve of these are positive regulators (secretion decreases when they are silenced), while seven are negative regulators (secretion increases when they are silenced). We likely did not identify several important regulators of this process either because in some cases the knockdown was not effective enough to reveal a phenotype or because we have silenced genes that are involved in constitutive splicing and are essential for cell viability. The identification of HMGB1, a well-known DAMP (Damage-associated molecular pattern) with a documented role in the activation of the IL-1β promoter [34], validates our capacity to discover essential regulators, even if HMGB1 is not a canonical splicing factor.

We chose SFRS3, a well characterized splicing factor that belongs to the SR family, mostly because the phenotype was very strong and consistent for 3 out of the 5 constructs used to target the gene, but also because it was a negative regulator of IL-1β secretion. The fact that the levels of SFRS3 are down-regulated in human monocytes by an *E. coli* challenge and that another member of the SR family of splicing factors identified in our screen is down-modulated in inflammatory conditions [38] points to these factors as important players in the modulation of IL-1β secretion and activity. Immune responsive cells have evolved negative regulatory mechanisms that function at multiple levels to effectively terminate an immune response and to prevent excessive host damage. However, the molecular mechanisms involved in terminating pro-inflammatory signals are incompletely understood and have been less explored. We therefore chose this strong negative regulator to further explore its mechanistic role in the regulation of IL-1β secretion.

While we still do not understand the fine molecular mechanisms that explain the role of SFRS3 in IL-1β secretion, it is clear that the phenotype results from a combination of the negative regulation in both IL-1β and caspase-1 mRNA levels which ultimately translates into significantly higher levels of pro-IL-1β and active caspase-1 to process it. By comparing the ratios of mRNA levels in SFRS3-silenced to control THP-1 cells, using either exonic or intronic primers directed to IL-1β, we could observe that they are similar, and therefore could conclude that transcription is the most affected step by silencing of SFRS3. To confirm that increased transcription is the dominant step responsible for the increased levels of IL-1β mRNA in SFRS3 silenced cells, we studied the stability of IL-1β in control and SFRS3 silenced cells after *E. coli* challenge and Actinomycin D treatment and found no significant differences in the half-life of IL-1β in both cases.

The transcription of IL-1β depends on the activation of the NF-κB pathway. Because its mRNA is increased in *E. coli* challenged THP-1 cells where SFRS3 was silenced, we hypothesized that the NF-κB pathway was more active in SFRS3 depleted cells. However, SFRS3 silencing does not change the expression of a GFP reporter under an NF-κB responsive promoter. This result suggests that other transcription factors that are known to have a role in IL-1β transcription, such as AP-1 or the more recently uncovered SORY and ATF7 transcription factors [40], are possible candidates to be affected by SFRS3.

The use of unbiased high-throughput methods such as splicing-sensitive arrays alone or in combination with RNAseq, to compare isoform representation in shRNA control or shRNA against SFRS3 treated cells, are likely to help identifying the molecular target(s) of SFRS3 which might be the direct effectors mediating the negative regulator phenotype of SFRS3 in the secretion of IL-1β. This line of research would be based on the assumption that SFRS3 is a key factor for the correct splicing of a direct negative regulator of IL-1β transcription.

In these study we have identified 19 novel regulators of IL-1β secretion, pointing to the relevance of splicing in the regulation of auto-inflammatory diseases, and other conditions that are associated with over-production of this pro-inflammatory cytokine. Further studies will determine the roles of SFRS3 and other factors in the control of IL-1β secretion.

## Supporting Information

Table S1 List of splicing factors tested for a role in IL-1β secretion using shRNA.
(DOCX)
