# Supplementary material for: An shRNA-Based Screen of Splicing Regulators Identifies SFRS3 as a Negative Regulator of IL-1β Secretion
Source: PLoS One. 2011 May 17;6(5):e19829. doi: 10.1371/journal.pone.0019829 (PMC3096647; doi:10.1371/journal.pone.0019829)
Supplement: Table S1 — List of splicing factors tested for a role in IL-1β secretion using shRNA. (DOCX) [file pone.0019829.s001.docx]

**An shRNA-based Screen of Splicing Regulators Identifies SFRS3 as a Negative Regulator of IL-1β Secretion**

Pedro M. Alves^1,^*, Ana Neves-Costa^1,^*, Helena Raquel^1,^*, Teresa Raquel Pacheco^1^, Bruno D’Almeida^1^, Raquel Rodrigues^1^, Iris Cadima-Couto^1^, Angelo Chora^1^, Mariana Oliveira^2^, Margarida Gama-Carvalho^2,3^, Nir Hacohen^4,5^ and Luis F. Moita^1,#^

^1^Instituto de Medicina Molecular, Faculdade de Medicina, Universidade de Lisboa, 1649-028 Lisboa, Portugal

^2^Centro de Biodiversidade, Genómica Funcional e Integrativa (BioFIG), Faculdade de Ciências, Universidade de Lisboa, 1749-016 Lisboa, Portugal

^3^Faculdade de Medicina, Universidade de Lisboa, 1649-028 Lisboa, Portugal

^4^Center for Immunology and Inflammatory Diseases Division of Rheumatology, Allergy, and Immunology Massachusetts General Hospital and Harvard Medical School Boston, MA 02129

^5^Broad institute of MIT and Harvard Cambridge, MA 02139

**Supplementary Table S1 - List of factors tested**

| **Gene Name** | **NM_Id** | **Gene Name** | **NM_Id** |
| --- | --- | --- | --- |
| **A2BP1** | NM_145891 | **NOVA1** | NM_006491 |
| **ABT1** | NM_013375 | **NOVA2** | NM_002516 |
| **ACIN1** | NM_014977 | **NUDT21** | NM_007006 |
| **ADAR** | NM_001111 | **NUMA1** | NM_006185 |
| **AKAP8** | NM_005858 | **NXF1** | NM_006362 |
| **AQR** | NM_014691 | **PABPC1** | NM_002568 |
| **ARS2** | NM_015908 | **PABPC4** | NM_003819 |
| **ASCC3L1** | NM_014014 | **PABPN1** | NM_004643 |
| **BAT1** | NM_004640 | **PARP1** | NM_001618 |
| **BCAS2** | NM_005872 | **PCBP1** | NM_006196 |
| **BRUNOL4** | NM_020180 | **PCBP2** | NM_005016 |
| **BRUNOL5** | NM_021938 | **PCBP3** | NM_020528 |
| **BRUNOL6** | NM_052840 | **PCBP4** | NM_020418 |
| **BUB3** | NM_004725 | **PHF5A** | NM_032758 |
| **C10orf116** | NM_006829 | **PIAS1** | NM_016166 |
| **C13orf10** | NM_022118 | **PLRG1** | NM_002669 |
| **C14orf166** | NM_016039 | **PNN** | NM_002687 |
| **C1orf55** | NM_152608 | **POLDIP3** | NM_032311 |
| **C1orf60** | NM_023015 | **POLDIP3** | NM_032311 |
| **C1QBP** | NM_001212 | **POLR2A** | NM_000937 |
| **C20orf14** | NM_012469 | **POLR2B** | NM_000938 |
| **C20orf23** | NM_024704 | **PPIE** | NM_006112 |
| **C21orf66** | NM_016631 | **PPIH** | NM_006347 |
| **C21orf70** | NM_058190 | **PPIL1** | NM_016059 |
| **C22orf19** | NM_003678 | **PPIL2** | NM_014337 |
| **C2orf3** | NM_003203 | **PPIL3** | NM_130906 |
| **C9orf10** | NM_014612 | **PPM1G** | NM_002707 |
| **CCDC12** | NM_144716 | **PPWD1** | NM_015342 |
| **CCNA1** | NM_003914 | **PRCC** | NM_005973 |
| **CCNK** | NM_003858 | **PRKRA** | NM_003690 |
| **CD2BP2** | NM_006110 | **PRPF18** | NM_003675 |
| **CDC2L2** | NM_024011 | **PRPF19** | NM_014502 |
| **CDC40** | NM_015891 | **PRPF3** | NM_004698 |
| **CDC5L** | NM_001253 | **PRPF31** | NM_015629 |
| **CHERP** | NM_006387 | **PRPF38A** | NM_032284 |
| **CIRBP** | NM_001280 | **PRPF38B** | NM_018061 |
| **CLK1** | NM_004071 | **PRPF4** | NM_004697 |
| **CLK2** | NM_003993 | **PRPF4B** | NM_003913 |
| **CLK3** | NM_003992 | **PRPF8** | NM_006445 |
| **CLK4** | NM_020666 | **PSEN1** | NM_000021 |
| **CPSF1** | NM_013291 | **PSIP1** | NM_021144 |
| **CPSF2** | NM_017437 | **PTBP1** | NM_002819 |
| **CPSF3** | NM_016207 | **PTBP2** | NM_021190 |
| **CPSF4** | NM_006693 | **QKI** | NM_006775 |
| **CPSF6** | NM_007007 | **RALY** | NM_016732 |
| **CRK7** | NM_016507 | **RAVER1** | NM_133452 |
| **CRNKL1** | NM_016652 | **RBBP7** | NM_002893 |
| **CSDA** | NM_003651 | **RBM10** | NM_005676 |
| **CSN3** | NM_005212 | **RBM12** | NM_006047 |
| **CSTF3** | NM_001326 | **RBM15** | NM_022768 |
| **CTNNBL1** | NM_030877 | **RBM17** | NM_032905 |
| **CUGBP1** | NM_006560 | **RBM22** | NM_018047 |
| **CUGBP2** | NM_006561 | **RBM25** | NM_021239 |
| **CWF19L1** | NM_018294 | **RBM3** | NM_006743 |
| **DDB1** | NM_001923 | **RBM5** | NM_005778 |
| **DDX1** | NM_004939 | **RBM7** | NM_016090 |
| **DDX17** | NM_006386 | **RBM8A** | NM_005105 |
| **DDX19B** | NM_007242 | **RBM9** | NM_014309 |
| **DDX21** | NM_004728 | **RBMS1** | NM_016836 |
| **DDX23** | NM_004818 | **RBMX** | NM_002139 |
| **DDX26** | NM_012141 | **RBMX2** | NM_016024 |
| **DDX39** | NM_005804 | **RBP7** | NM_052960 |
| **DDX3X** | NM_001356 | **RDBP** | NM_002904 |
| **DDX41** | NM_016222 | **REXO1** | NM_020695 |
| **DDX46** | NM_014829 | **REXO2** | NM_015523 |
| **DDX48** | NM_014740 | **RKHD3** | NM_032246 |
| **DDX49** | NM_019070 | **RNGTT** | NM_003800 |
| **DDX5** | NM_004396 | **RNPC2** | NM_004902 |
| **DDX54** | NM_024072 | **RNPS1** | NM_006711 |
| **DDX6** | NM_004397 | **ROD1** | NM_005156 |
| **DEK** | NM_003472 | **RP13-297E16.1** | NM_005088 |
| **DGCR14** | NM_022719 | **RPL22** | NM_000983 |
| **DHX15** | NM_001358 | **RPL23A** | NM_000984 |
| **DHX16** | NM_003587 | **RPL31** | NM_000993 |
| **DHX35** | NM_021931 | **RPL5** | NM_000969 |
| **DHX38** | NM_014003 | **RPS10** | NM_001014 |
| **DHX8** | NM_004941 | **RPS11** | NM_001015 |
| **DHX9** | NM_001357 | **RPS12** | NM_001016 |
| **DIDO1** | NM_022105 | **RPS13** | NM_001017 |
| **DKFZP434K1421** | NM_032141 | **RPS15** | NM_001018 |
| **DNAJC17** | NM_018163 | **RPS15A** | NM_001019 |
| **DNAJC6** | NM_014787 | **RPS16** | NM_001020 |
| **DNAJC8** | NM_014280 | **RPS17** | NM_001021 |
| **EDG2** | NM_057159 | **RPS18** | NM_022551 |
| **EEF1A1** | NM_001402 | **RPS19** | NM_001022 |
| **EFTUD2** | NM_004247 | **RPS25** | NM_001028 |
| **EIF2S2** | NM_003908 | **RPS29** | NM_001032 |
| **EIF3S10** | NM_003750 | **RPS3** | NM_001005 |
| **EIF3S2** | NM_003757 | **RPS3A** | NM_001006 |
| **EIF3S6** | NM_001568 | **RPS4X** | NM_001007 |
| **EIF3S6IP** | NM_016091 | **RPS4Y1** | NM_001008 |
| **EIF4A2** | NM_001967 | **RPS5** | NM_001009 |
| **ELAVL1** | NM_001419 | **RPS7** | NM_001011 |
| **ELAVL2** | NM_004432 | **RPS8** | NM_001012 |
| **ELAVL3** | NM_001420 | **RPS9** | NM_001013 |
| **ELAVL4** | NM_021952 | **RUVBL1** | NM_003707 |
| **EP400** | NM_015409 | **RUVBL2** | NM_006666 |
| **ERCC3** | NM_000122 | **RY1** | NM_006857 |
| **ERVWE1** | NM_014590 | **S100A8** | NM_002964 |
| **ET** | NM_024311 | **S100A9** | NM_002965 |
| **EWSR1** | NM_005243 | **SAFB** | NM_002967 |
| **EXOSC1** | NM_016046 | **SAFB2** | NM_014649 |
| **EXOSC10** | NM_002685 | **SART1** | NM_005146 |
| **EXOSC2** | NM_014285 | **SDCCAG10** | NM_005869 |
| **EXOSC3** | NM_001002269 | **SF1** | NM_004630 |
| **EXOSC4** | NM_019037 | **SF3A1** | NM_005877 |
| **EXOSC5** | NM_020158 | **SF3A2** | NM_007165 |
| **EXOSC7** | NM_015004 | **SF3A3** | NM_006802 |
| **EXOSC8** | NM_181503 | **SF3B1** | NM_012433 |
| **EXOSC9** | NM_005033 | **SF3B14** | NM_016047 |
| **FAM32A** | NM_014077 | **SF3B3** | NM_012426 |
| **FAU** | NM_001997 | **SF3B4** | NM_005850 |
| **FIP1L1** | NM_030917 | **SF3B5** | NM_031287 |
| **FKBP3** | NM_002013 | **SF4** | NM_021164 |
| **FLJ20273** | NM_019027 | **SFPQ** | NM_005066 |
| **FLJ21827** | NM_020153 | **SFRS1** | NM_006924 |
| **FMR1** | NM_002024 | **SFRS10** | NM_004593 |
| **FRG1** | NM_004477 | **SFRS11** | NM_004768 |
| **FUBP1** | NM_003902 | **SFRS12** | NM_139168 |
| **FUS** | NM_004960 | **SFRS16** | NM_007056 |
| **FUSIP1** | NM_006625 | **SFRS2** | NM_003016 |
| **G10** | NM_003910 | **SFRS3** | NM_003017 |
| **GNB2L1** | NM_006098 | **SFRS4** | NM_005626 |
| **GPATC1** | NM_018025 | **SFRS5** | NM_006925 |
| **GRSF1** | NM_002092 | **SFRS6** | NM_006275 |
| **GTF2I** | NM_001518 | **SFRS7** | NM_006276 |
| **GTL3** | NM_013242 | **SFRS9** | NM_003769 |
| **HCFC1** | NM_005334 | **SIAHBP1** | NM_014281 |
| **HDAC2** | NM_001527 | **SKIV2L** | NM_006929 |
| **HIST1H2AC** | NM_003512 | **SKIV2L2** | NM_015360 |
| **HIST1H2BC** | NM_003526 | **SLU7** | NM_006425 |
| **HIST2H2AA** | NM_003516 | **SMARCA5** | NM_003601 |
| **HMGB1** | NM_002128 | **SMC1L1** | NM_006306 |
| **HMGB3** | NM_005342 | **SMC2L1** | NM_006444 |
| **HNRNPG-T** | NM_014469 | **SMNDC1** | NM_005871 |
| **HNRPA0** | NM_006805 | **SMU1** | NM_018225 |
| **HNRPA1** | NM_002136 | **SNIP1** | NM_024700 |
| **HNRPA2B1** | NM_002137 | **SNRP70** | NM_003089 |
| **HNRPA3** | NM_194247 | **SNRPA** | NM_004596 |
| **HNRPAB** | NM_004499 | **SNRPA1** | NM_003090 |
| **HNRPC** | NM_031314 | **SNRPB** | NM_198216 |
| **HNRPCL1** | NM_001013631 | **SNRPB2** | NM_198220 |
| **HNRPD** | NM_031370 | **SNRPC** | NM_003093 |
| **HNRPDL** | NM_005463 | **SNRPD1** | NM_006938 |
| **HNRPF** | NM_004966 | **SNRPD2** | NM_004597 |
| **HNRPH1** | NM_005520 | **SNRPD3** | NM_004175 |
| **HNRPH2** | NM_019597 | **SNRPE** | NM_003094 |
| **HNRPH3** | NM_012207 | **SNRPF** | NM_003095 |
| **HNRPK** | NM_002140 | **SNRPG** | NM_003096 |
| **HNRPL** | NM_001533 | **SNRPN** | NM_003097 |
| **HNRPLL** | NM_138394 | **SNW1** | NM_012245 |
| **HNRPM** | NM_005968 | **SPEN** | NM_015001 |
| **HNRPR** | NM_005826 | **SPPL3** | NM_139015 |
| **HNRPU** | NM_031844 | **SR140** | XM_031553 |
| **HNRPUL1** | NM_007040 | **SRP19** | NM_003135 |
| **HSPA1B** | NM_005346 | **SRP46** | NM_032102 |
| **HSPA5** | NM_005347 | **SRP68** | NM_014230 |
| **HSPA8** | NM_006597 | **SRP9** | NM_003133 |
| **HSPC117** | NM_014306 | **SRPK1** | NM_003137 |
| **HSPC148** | NM_016403 | **SRRM1** | NM_005839 |
| **HTATSF1** | NM_014500 | **SRRM2** | NM_016333 |
| **HYPC** | NM_012272 | **SSB** | NM_003142 |
| **IK** | NM_006083 | **STK23** | NM_014370 |
| **ILF2** | NM_004515 | **STRBP** | NM_018387 |
| **ILF3** | NM_004516 | **SYF2** | NM_015484 |
| **IMP-1** | NM_006546 | **SYNCRIP** | NM_006372 |
| **IMP-3** | NM_006547 | **TAF15** | NM_003487 |
| **IQGAP1** | NM_003870 | **TAF6** | NM_005641 |
| **KHSRP** | NM_003685 | **TCERG1** | NM_006706 |
| **KIAA0773** | NM_014690 | **TCERG1** | NM_006706 |
| **KIAA1008** | NM_014953 | **TDRD3** | NM_030794 |
| **KIAA1160** | NM_020701 | **TET1** | NM_030625 |
| **KIAA1429** | NM_015496 | **TFIP11** | NM_001008697 |
| **KIAA1542** | NM_020901 | **THOC1** | NM_005131 |
| **KIAA1604** | NM_020943 | **THOC3** | NM_032361 |
| **KIAA1967** | NM_021174 | **THOC4** | NM_005782 |
| **KIN** | NM_012311 | **TIA1** | NM_022173 |
| **KPNA2** | NM_002266 | **TIAL1** | NM_003252 |
| **LOC138046** | NM_173848 | **TNPO1** | NM_002270 |
| **LSM1** | NM_014462 | **TNRC4** | NM_007185 |
| **LSM10** | NM_032881 | **TOP1MT** | NM_052963 |
| **LSM11** | NM_173491 | **TOPORS** | NM_005802 |
| **LSM2** | NM_021177 | **TPR** | NM_003292 |
| **LSM3** | NM_014463 | **TPX2** | NM_012112 |
| **LSM4** | NM_012321 | **TRA2A** | NM_013293 |
| **LSM5** | NM_012322 | **TRNT1** | NM_016000 |
| **LSM6** | NM_007080 | **TTF2** | NM_003594 |
| **LSM7** | NM_016199 | **TUBA1** | NM_006000 |
| **LSM8** | NM_016200 | **TUBB** | NM_178014 |
| **LUC7L** | NM_018032 | **TXNL4A** | NM_006701 |
| **MAGOH** | NM_002370 | **U2AF1** | NM_006758 |
| **MATR3** | NM_018834 | **U2AF1L2** | NM_005089 |
| **MBD5** | NM_018328 | **U2AF1L3** | NM_144987 |
| **MFAP1** | NM_005926 | **U2AF2** | NM_007279 |
| **MGC13125** | NM_032725 | **UBL5** | NM_024292 |
| **MGC14151** | NM_032356 | **UNK** | NM_152302 |
| **MGC2803** | NM_024038 | **USP39** | NM_006590 |
| **MGC5509** | NM_024093 | **VIM** | NM_003380 |
| **MKI67IP** | NM_032390 | **WBP11** | NM_016312 |
| **MORG1** | NM_032332 | **WDR33** | NM_001006622 |
| **MOV10** | NM_020963 | **WDR57** | NM_004814 |
| **MSI1** | NM_002442 | **WDR58** | NM_024339 |
| **MSI2** | NM_138962 | **WTAP** | NM_004906 |
| **MYEF2** | NM_016132 | **XAB2** | NM_020196 |
| **NCBP1** | NM_002486 | **XRCC6** | NM_001469 |
| **NCBP2** | NM_007362 | **XRN2** | NM_012255 |
| **NCL** | NM_005381 | **YBX1** | NM_004559 |
| **NDUFA1** | NM_004541 | **ZC3H13** | NM_015070 |
| **NHN1** | NM_144604 | **ZCCHC8** | NM_017612 |
| **NHP2L1** | NM_001003796 | **ZFP36L1** | NM_004926 |
| **NIF3L1BP1** | NM_025075 | **ZFR** | NM_016107 |
| **NONO** | NM_007363 | **ZMAT2** | NM_144723 |
| **NOSIP** | NM_015953 | **ZNF207** | NM_003457 |
